# Editorial: Surgical experience and complications are inextricably linked

**DOI:** 10.3389/fsurg.2023.1253985

**Published:** 2023-08-02

**Authors:** Philippe Sebe

**Affiliations:** Department of Urology, University Hospitals of Geneva, Geneva, Switzerland

**Keywords:** robotic surgery, pelvic surgery, surgical outcomes, complications, perioperative morbidity

**Editorial on the Research Topic** Surgical experience and complications are inextricably linked

Surgical complications play a major role in the life of any surgeon, whatever their specialty. At best, complications keep you awake at night, at worst they can land you in court. We are proud to play our part in helping to manage them. Post-operative medicine is rarely taught at university, even though it has many specific features in terms of diagnosis and treatment. What's more, the importance of complications is often underestimated in the medical literature, which favors positive results. That's why this research topic dedicated to the complications of minimally invasive surgery is so important to us. It also looks at complications from a preventive point of view, providing tips and tricks. Minimally invasive robotic-assisted pelvic surgery is in full expansion, and we feel it is important to accompany its development by proposing articles prospectively documenting the short- and long-term post-operative evolution of these complex surgeries.

In this context of therapeutic innovation, it is vital to publish articles whose data reflect our day-to-day reality, particularly in the field of functional results of radical prostatectomy. Very early in my career, I learned that the functional results of radical prostatectomy were often magnified, and that their veracity was one of the taboos of urology. How many articles were published with functional results that gave the operated population a continence and sexual potency superior to the non-operated population of the same age? To illustrate this point, Alessio Paladini's team has aptly examined the functional results of robot-assisted laparoscopic radical prostatectomy (Paladini et al.). Urinary continence and sexual potency were restored in 49.1%, 66.7% and 79.6% of patients, and in 19.1%, 29.9% and 36.2% of patients at 3, 6 and 12 months, respectively. The indications were high-risk prostate cancers and the technique an extra-fascial dissection without preservation of the neuro-vascular bundles. Therefor the results are in line with experience of non-conservative surgery. In the pre-operative information that will be delivered to patients, these data are of great value, as patients expect minimally invasive surgery to deliver near-perfect functional results. Moreover, for teams new to minimally invasive surgery, it is vital to be able to draw on such results to assess their initial experience, and to set medium-term functional outcome targets, which will initially depend on the learning curve.

The same team has also published a high-quality work on robot-assisted laparoscopic cystectomy (Cochetti et al.). This procedure is currently the most complex in minimally invasive urological surgery, since it includes extensive pelvic lymph node dissections, total cystectomy and reconstruction of the lower urinary tract using an ileal bladder or Bricker-type ileal derivation. In the early stages of experience, operating times are long, and in my opinion it is difficult to start with fully robotic procedures. An alternative might be to perform the reconstruction via an open approach, followed by robotic reconstruction when lymphadenectomy and cystectomy are routine. When deciding to carry out the procedure completely robotically, it is therefore important to have operative time objectives in mind, so as not to increase perioperative morbidity. In this respect, the reported work is very interesting, since it provides a precise estimate of perioperative morbidity when the operative time is about 4 to 6 h, which is a reasonable objective.

These articles are of great pedagogical interest, as they faithfully report on a highly complex robotic pelvic surgery experience, and are a great help to the development of these innovative minimally invasive techniques.

